# HER2‐Ultralow: Prevalence, Characteristics, and Treatment Choices Among Advanced Breast Cancer Patients With Tumors Initially Scored as IHC 0

**DOI:** 10.1155/tbj/7212120

**Published:** 2026-06-24

**Authors:** Anushree R. Iyengar, Erinn P. Downs, Sandhya Mehta, Nivedita Rangarajan, Michele Sue-Ann Woo, Simone T. Sredni, Rosemarie Di Donato, Safak Simsek, Darren M. Wilson, Katherine Krieser, Elise Bieri Patzke, Natalie Kyek, Christian Anderson, Tyler Wagner, Jason Hipp, Aziza Nassar, Hannah Barman

**Affiliations:** ^1^ Department of Biomedical Research, Nference, Inc., Cambridge, Massachusetts, USA; ^2^ Department of Laboratory Medicine and Pathology, Mayo Clinic, Scottsdale, Arizona, USA, mayo.edu; ^3^ US Medical Affairs, Daiichi Sankyo, Inc., Basking Ridge, New Jersey, USA; ^4^ Biomarker Science and Technology, AstraZeneca, LLC, Gaithersburg, Maryland, USA; ^5^ Department of Laboratory Medicine and Pathology, Mayo Clinic, Rochester, Minnesota, USA, mayo.edu; ^6^ BioPharma Diagnostics, Mayo Clinic, Rochester, Minnesota, USA, mayo.edu; ^7^ Clinical Research Operations, Mayo Clinic, Rochester, Minnesota, USA, mayo.edu; ^8^ Mayo Clinic Comprehensive Cancer Center, Mayo Clinic, Jacksonville, Florida, USA, mayo.edu

**Keywords:** breast cancer, HER2, immunohistochemistry

## Abstract

Historically, HER2 status in invasive breast cancer has been categorized as HER2‐positive (IHC 3+, IHC 2+/ISH+) or HER2‐negative (IHC 0, IHC 1+, IHC 2+/ISH‐). Patients meeting IHC 0 with membrane staining (HER2‐ultralow) criteria may benefit from HER2‐targeted therapies such as trastuzumab deruxtecan. This cohort study assessed the prevalence of HER2‐ultralow expression by re‐scoring HER2 IHC slides using Mayo Clinic electronic health record data. Three hundred patients with advanced breast cancer (Stages III‐IV) and documented HER2 IHC 0 status (January 2017–March 2023) were identified. One slide per patient was digitized and independently re‐scored by two Mayo Clinic pathologists following the 2023 ASCO‐CAP guidelines, including tumor staining percentage to denote HER2‐ultralow status. A sensitivity analysis was performed by a third independent pathologist. The re‐scored patients had a mean age of 57.7 years (SD = 13.6). Most samples (95%, *n* = 285) remained scored IHC 0 by at least one pathologist; 60% of these met HER2‐ultralow criteria per at least one pathologist. HER2‐ultralow prevalence ranged from 43% to 45% per pathologist, with 57% overall interpathologist concordance. Samples with no observable staining comprised most of the concordant cases. Treatment patterns were similar between HER2‐ultralow and no‐staining groups; however, time to treatment failure (TTF) varied between groups across lines of therapy (LOT). In HR− positive cohorts, median TTF for LOT1 was 7.73 months in patients with HER2‐ultralow versus 9.43 months with no observable IHC staining. In HR− negative cohorts, median TTF was 5.00 months for HER2‐ultralow versus 3.17 months with no observable IHC staining. Similar TTF trends were observed in LOT2‐LOT3. Approximately three in five samples originally classified as IHC 0 met HER2‐ultralow criteria, suggesting many patients may benefit from HER2‐directed therapy if reclassified. These findings highlight potential challenges of identifying HER2‐ultralow expression and suggest the need for enhanced pathologist training, adherence to best practices, and integration of digital pathology and artificial intelligence solutions.

**Trial Registration:** ClinicalTrials.gov_identifier: NCT03734029

## 1. Introduction

Invasive breast cancer (BC) biomarker testing typically includes immunohistochemistry (IHC) for human epidermal growth factor 2 (HER2 or ERBB2) expression. HER2 IHC results are reported in a binary classification of positive (IHC 3+) versus negative (IHC 0 or IHC 1+); samples that are IHC 2+ require additional evaluation with in situ hybridization (ISH) and may ultimately be either IHC 2+/ISH positive (ISH+) or IHC 2+/ISH negative (ISH‐).

The DESTINY‐Breast04 clinical trial demonstrated the efficacy of trastuzumab deruxtecan (T‐DXd), a HER2‐directed antibody‐drug conjugate (ADC), in patients with HER2‐low metastatic BC (mBC), defined as IHC 1+ or IHC 2+/ISH− [1]. The recent US approval of T‐DXd for the treatment of HER2‐low (IHC 1+ or IHC 2+/ISH‐) mBC based on these trial results is leading to reconsideration of the HER2 spectrum framework to include HER2‐positive (IHC 2+/ISH + or IHC 3+), HER2‐low (IHC 1+ or IHC 2+/ISH‐), and IHC 0 disease. An additional subclassification within IHC 0 has been recently recognized: HER2‐ultralow, for which T‐DXd is now approved, describes tumors with incomplete HER2 membrane staining that is faint and barely perceptible in ≤ 10% of tumor cells (IHC 0 with membrane staining) [2].

About 50% of the BC patients in the US who were traditionally classified as having HER2‐negative BC have HER2‐low tumors, and 65%–85% of these patients are hormone receptor (HR) positive (HR+) [3–5]. DESTINY‐Breast06 is a Phase 3 clinical trial evaluating the efficacy, safety, and tolerability of T‐DXd compared with investigator’s choice chemotherapy in HER2‐low and HER2‐ultralow HR+ mBC [2]. Data from the primary trial analysis show that T‐DXd is associated with improved median progression‐free survival (PFS) compared to chemotherapy in both HER2‐low (13.2 vs. 8.1 months) and HER2‐ultralow (13.2 vs. 8.3 months) HR+ mBC patients [2].

As T‐DXd is now an approved treatment option for HER2‐low and ‐ultralow mBC, it is crucial to determine the US prevalence of HER2‐ultralow for personalized treatment strategies. The present study used retrospective electronic health record (EHR) data from Mayo Clinic to assess the prevalence of HER2‐ultralow in patients with advanced BC (aBC) or mBC (aBC) with a historical IHC 0 score, based on digitized biopsy slides reviewed by expert pathologists. Findings from this study provide insights on the prevalence and unmet needs of HER2‐ultralow disease and are expected to assist healthcare decision‐makers for the appropriate management of aBC across the HER2 spectrum.

## 2. Methods

### 2.1. Cohort Creation

A retrospective cohort study was conducted for aBC patients who had HER2‐stained tissue biopsies documented as IHC 0 at Mayo Clinic between 2017–2023. Of all aBC patients with available biopsies, the most recent biopsy slides from any local or metastatic tissue/site (one per patient) for the 300 most recent patients were selected for analysis. Although the DESTINY‐Breast06 trial includes only patients with mBC [2], both advanced and metastatic cases were included in this study due to sample size requirements.

### 2.2. Identification of aBC Patients

BC patients were identified using *International Classification of Diseases, Ninth Revision (ICD-9); International Classification of Diseases, Tenth Revision* (*ICD-10*); and Systematized Nomenclature of Medicine codes, along with a natural language processing (NLP) algorithm (“disease diagnosis model”) [6–8] applied to clinical notes mentioning BC. The model, trained on 40,000 tagged sentences with a 70:30% train/test split, confirmed BC diagnosis for patients with sentences labeled as “Yes” with at least 0.8 confidence [9]. To identify aBC patients, a second NLP algorithm (“staging model”) [6–8] was used on clinical notes to assign cancer stages. This model, trained on 18,700 tagged sentences with an 80:20% train/test split, categorized stages as “early,” “advanced,” or “metastatic” based on clinical and TNM stages. Secondary neoplasm *ICD-10* codes were used to detect undocumented metastatic sites, with lymph node involvement classified as advanced and other viscera as metastatic (see Supporting Tables S1‐S2).

### 2.3. Identification of HER2 IHC Scoring

Patients were required to have prior documentation of a HER2 IHC 0 score to be eligible for inclusion. A Regular Expression (RegEx) module was developed to extract IHC scores (0–3+) from all clinical notes, including pathology reports. RegEx rules were based on manual review of 500 sentences containing mention of IHC scores from pathology reports. The module was validated on 1644 sentences and achieved near‐perfect performance with an F1 score of 1.00 (Supporting Table S3). This module was run on clinical notes of patients with valid HR statuses.

Institutional review board approval was obtained to query the Laboratory Information System across the Mayo Clinic Enterprise (Minnesota, Arizona, and Florida) for Mayo Clinic patient cases with characterized aBC, with tissue samples from either the primary or metastatic lesion, with an IHC 0 score. The three hundred most recent previously signed‐out cases were identified with the search criteria above from consented donors between January 2017 and March 2023 and confirmed with pathological diagnosis and case chart review.

All qualifying pathology slides were digitized at the Mayo digital pathology scanning facility using Pramana’s (Cambridge, MA, USA) high‐throughput digital scanners. The images were linked to the patient’s de‐identified medical records, enabling review by three different pathologists. Each slide was scanned at 40X magnification and 0.26 µm/pixel scan resolution, leveraging dynamic Z‐stacks of each high‐power field for real‐time focal quality assessment and continual refinement, which allows for in‐line quality assurance and autonomous re‐scanning as needed. Any deficiencies such as out‐of‐focus fields, stitching errors, and other detectable errors were detected, displayed to the users, and saved as metadata. Once the digital images passed quality control, they were converted into a DICOM object with visually lossless compressed JPEG pixel data and transferred to the Mayo Cloud platform.

Images were reviewed by two board‐certified pathologists from Mayo Clinic who were blinded to previous interpretations of each specimen, to each other’s interpretations, and to the goal of identifying HER2‐ultralow cases. The pathologists scored the retrospective de‐identified digital images in accordance with the most recent 2023 ASCO/CAP guidelines [10]. A scoring sheet was developed to capture the IHC score (0–3+, or “indeterminate” in the case of issues with sample or image quality). Each pathologist independently completed one score sheet per case. For IHC 0 cases, the percentage of tumor cell staining (0%–10%) was also captured [9]; patients were considered HER2‐ultralow if membrane staining was observable or “no observable IHC staining” if no membrane staining was observable on the slide. The results of both pathologists’ reviews were aggregated. The full analysis cohort consisted of patients classified as IHC 0 by either pathologist; patients with 1%–10% of recorded tumor staining by either pathologist were considered HER2‐ultralow patients, while the remaining were classified as having no observable IHC membrane staining. Patients scored as IHC 1+, 2+, or 3+ by either pathologist were excluded from downstream analyses.

As a sensitivity analysis to further assess HER2‐ultralow prevalence and concordance between pathologists, a third board‐certified general pathologist from nference re‐scored all biopsies in the same manner as the previous two pathologists. Patients were then classified based on agreement between at least two of the three pathologists.

#### 2.3.1. Patient Clinical Characteristics

Estrogen receptor (ER) and progesterone receptor (PgR) statuses for aBC patients were determined using an NLP algorithm trained on 28,700 manually tagged sentences from Mayo EHR with an 80:20% train/test split (see metrics in Supporting Table S3) [6–8]. Sentences were labeled “Positive” or “Negative” by the model; sentences were considered in assigning ER/PgR statuses if the label confidence was at least 0.8. The model ran in three iterations, covering synonyms for “estrogen receptor,” “progesterone receptor,” and “hormone receptor.” For each patient, biomarker results closest to their staging date were used. Patients were classified as HR+ if either ER, PgR, or HR was positive. If both ER and PgR were negative or HR was negative, patients were considered HR− negative (HR−). Metastasis locations and counts were assessed using *ICD-10* codes for secondary neoplasms (Supporting Table S1).

#### 2.3.2. Treatment Patterns and Outcomes

Lines of therapy (LOT) were identified for patients with HER2‐ultralow or no observable IHC staining who received an anticancer drug (Supporting Table S4) on or after their staging date. A patient’s first LOT (LOT1) started on the date of their first anticancer drug order/administration at Mayo after their staging date and encompassed any other anticancer drugs ordered/administered within 28 days of the LOT start. The length of each LOT was defined as the time between the first and last drug orders plus 21 days, to account for the full duration of the last prescription. Patients were considered to progress to their next LOT if a new anticancer drug was ordered/administered after LOT start, if there was a treatment gap of at least 360 days for regimens containing oral drugs, or if there was a treatment gap of at least 180 days for regimens containing only parenteral drugs. However, a patient’s LOT did not advance (i.e., considered as continuation of the same LOT) if there was a switch between docetaxel and paclitaxel, between platinum‐based chemotherapies, or between letrozole, anastrozole, and exemestane. Additionally, if a patient started docetaxel or paclitaxel within 100 days after starting a regimen containing cyclophosphamide and doxorubicin, these drugs were all considered as part of the same LOT. Patients were followed using data from their Mayo Clinic EHR through a maximum of four LOTs. For simplicity in reporting, regimens were grouped into categories, as detailed in Supporting Table S5.

Time to treatment failure (TTF), defined as time from LOT start to discontinuation or death, was assessed for patients with at least one LOT. Patients were censored at the LOT end date for a given LOT if their last encounter at Mayo was within 120 days of LOT end, as they were considered to have remained on treatment. For patients who proceeded to a new LOT or ended their LOT but had more than 120 days of subsequent follow‐up at Mayo with no further treatment (discontinuation), the date of treatment failure was defined as the LOT end date (i.e., last drug order date plus 21 days). However, for patients who were on a LOT with only one medication order or administration and then proceeded to the next LOT, the date of treatment failure was instead the start date of the next LOT, assuming that they continued their original LOT outside of Mayo. If a patient died within 21 days of their last drug order date in a LOT, their date of treatment failure was defined as their date of death.

#### 2.3.3. Statistical Analyses

Patients’ clinicopathologic characteristics (e.g., demographics, HR status, sites of metastases) were summarized through descriptive analysis. Interpathologist concordance for scoring cases was calculated using Fleiss’ kappa (*κ*), which assesses inter‐rater agreement across two or more categories among two or more raters. Fleiss’ *κ* was calculated encompassing all four categories (no observable IHC staining, HER2‐ultralow, IHC 1+, IHC 2+/indeterminate) separately, as well as for each category as a binary (e.g., HER2‐ultralow vs. not HER2‐ultralow). Fleiss’ *κ* was interpreted as follows: *κ* < 0 suggests poor agreement; 0 ≤ *κ* ≤ 0.20 suggests slight agreement; 0.21 ≤ *κ* ≤ 0.40 suggests fair agreement; 0.41 ≤ *κ* ≤ 0.60 suggests moderate agreement; 0.61 ≤ *κ* ≤ 0.80 suggests substantial agreement; and 0.81 ≤ *κ* ≤ 1 suggests almost perfect agreement [11].

For patients receiving cancer treatment at Mayo, LOTs were aggregated by regimen class and summarized descriptively. Median time‐to‐event (i.e., time at which 50% of the cohort experienced an event) and 95% confidence intervals were derived using Kaplan–Meier estimators. Differences in clinical outcomes were analyzed using log‐rank tests. Analyses were stratified by HER2 classification and HR status.

## 3. Results

### 3.1. Cohort Identification and Creation

Based on the combination of diagnosis codes and NLP approaches, we identified 5091 aBC patients whose tumors were previously scored as HER2 IHC 0. The 300 patients included in the pathologist’s re‐scoring were the most recent cases from this set of patients.

### 3.2. Pathologist Concordance

Results of the pathologists’ review are summarized in Table 1. Of the 300 tumor samples, 285 (95%) remained classified as IHC 0 (either HER2‐ultralow or no observable staining) by at least one pathologist. Of these, 171 (60%) were classified as HER2‐ultralow by at least one pathologist. Per individual pathologist review, the prevalence of HER2‐ultralow among confirmed IHC 0 patients ranged from 43.1% (Pathologist 2; 121/281) to 44.6% (Pathologist 1; 104/233). The pathologists demonstrated moderate agreement in scoring no observable IHC staining (Fleiss’ *κ* *= *0.4593) and IHC 2+/indeterminate (*κ* *= *0.5664) patients, but only slight agreement in classifying HER2‐ultralow (*κ* *= *0.1680) and IHC 1+ (*κ* *= *0.1691). Pathologist 1 had a higher rate of IHC 1+ scores, whereas Pathologist 2 had a higher rate of HER2‐ultralow classifications. Overall, the pathologists exhibited fair agreement (*κ* *= *0.2950), showing full agreement on the classification of 171 patients across HER2 IHC scores (57%).

**TABLE 1:** Results of pathologists’ re‐scoring.

HER2 score	Individual pathologist results (*n* = 300 with historical IHC 0 score)		Concordance		
	Pathologist 1	Pathologist 2	1 of 2 pathologists	2 of 2 pathologists	Fleiss *κ*
No observable IHC staining	129	160	185	104	0.4593
HER2‐ultralow	104	121	171	54	0.1680
IHC 1+	62	17	68	11	0.1691
IHC 2+/indeterminate	5	2	5	2	0.5664
			Total Concordant	**171**	**0.2950**

*Note:* HER2, human epidermal growth factor 2; IHC, immunohistochemistry. Overall, the pathologists exhibited fair agreement (κ = 0.2950), with total concordance on the classification of 171 patients across HER2 IHC scores (57%).

Samples classified as HER2‐ultralow by either pathologist were considered as HER2‐ultralow, while all other IHC 0 samples were considered as no observable IHC staining. Based on this approach, we identified 171 patients in the HER2‐ultralow cohort and 114 patients in the no observable IHC staining cohort for treatment patterns and clinical outcomes analysis.

### 3.3. Sensitivity Analysis

**FIGURE 1:**
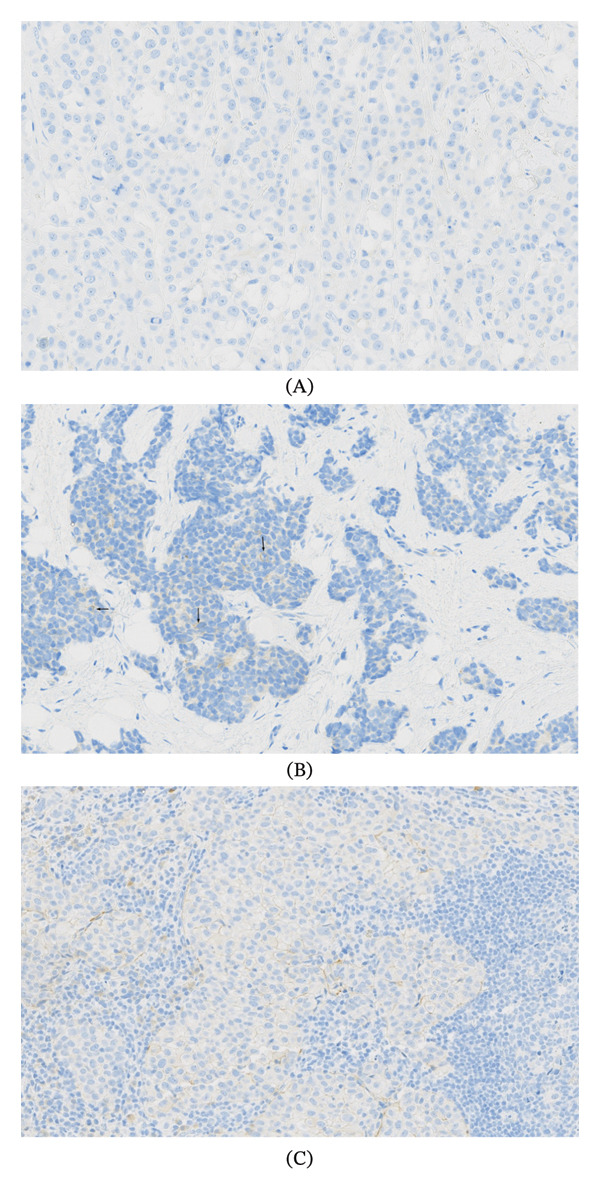
Slides unanimously scored by all 3 pathologists as (A) having no observable staining; (B) HER2‐ultralow (annotated staining with arrows); and (C) IHC 1+.

Table 2 summarizes the results of a sensitivity analysis performed by adding a third pathologist to re‐score the same slides. The prevalence of HER2‐ultralow among IHC 0 patients, according to this pathologist, was 56.3%. With the addition of a third pathologist, the level of interobserver agreement increased from slight to fair for HER2‐ultralow (*κ* *= *0.2720) and IHC 1+ (*κ* *= *0.3168). Agreement in classifying cases with no observable IHC staining remained stable. In total, two of three pathologists agreed in classifying 291 cases (97%), and all three pathologists agreed in classifying 129 cases (43%), resulting in fair agreement overall (*κ* *= *0.3618). Figure 1 presents representative samples that were classified as no observable IHC staining, HER2‐ultralow, or IHC 1+ concordantly by all pathologists.

**TABLE 2:** Sensitivity analysis for pathologist re‐scoring.

HER2 score	Individual pathologist results (*n* = 300 with historical IHC 0 score)			Concordance			
	Pathologist 1	Pathologist 2	Pathologist 3	1 of 3 pathologists	2 of 3 pathologists	3 of 3 pathologists	Fleiss *κ*
No observable IHC staining	129	160	115	196	131	77	0.4655
HER2‐ultralow	104	121	148	202	128	43	0.2720
IHC 1+	62	17	33	75	29	8	0.3168
IHC 2+/indeterminate	5	2	4	7	3	1	0.4478
				Total Concordant	**291**	**129**	**0.3618**

*Note:* HER2, human epidermal growth factor 2; IHC, immunohistochemistry. Two of three pathologists agreed in classifying 291 cases (97%), and all three pathologists agreed in classifying 129 cases (43%), resulting in fair agreement overall (κ = 0.3618).

### 3.4. Cohort Characteristics

The clinicopathological characteristics of the full cohort, in addition to the HER2‐ultralow and no observable IHC staining cohorts, are reported in Table 3. The median age of the full cohort was 57.7 years; the HER2‐ultralow cohort had a slightly lower median age compared to the no observable IHC staining cohort (57.0 vs. 58.8 years, respectively). All cohorts were predominantly White, non‐Hispanic/Latino, Midwestern females. The full cohort had primarily mBC with HR+ status. The no observable IHC staining cohort had a higher proportion of mBC (76% vs. 72%) and HR− (41% vs. 36%) cases compared with the HER2‐ultralow cohort.

**TABLE 3:** Demographic and clinical characteristics for full, HER2‐ultralow, and no observable IHC staining cohorts.

	**All re-scored patients (*n* = 300)**	**HER2-ultralow (*n* = 171)**	**No observable IHC staining (*n* = 114)**

Age at aBC index, mean (SD), years	57.80 (13.60)	57.11 (12.80)	58.76 (14.66)
Sex, *n* (%)			
Female	299 (99.67)	170 (99.42)	114 (100.00)
Male	1 (0.33)	1 (0.58)	0 (0.00)
Race, *n* (%)			
Asian	6 (2.00)	3 (1.75)	3 (2.63)
Black or African American	13 (4.33)	9 (5.26)	2 (1.75)
Other/unknown	15 (5.00)	6 (3.51)	9 (7.89)
White	266 (88.67)	153 (89.47)	100 (87.72)
Ethnicity, *n* (%)			
Hispanic or Latino	6 (2.00)	3 (1.75)	2 (1.75)
Not Hispanic or Latino	285 (95.00)	162 (94.74)	109 (95.61)
Other/unknown	9 (3.00)	6 (3.51)	3 (2.63)
Region, *n* (%)			
Northeast	7 (2.33)	3 (1.75)	3 (2.63)
Midwest	216 (72.00)	122 (71.35)	82 (71.93)
South	52 (17.33)	30 (17.54)	20 (17.54)
West	25 (8.33)	16 (9.36)	9 (7.89)
Unknown	0 (0.00)	0 (0.00)	0 (0.00)
Disease stage, *n* (%)			
Advanced	83 (27.67)	48 (28.07)	27 (23.68)
Metastatic	217 (72.33)	123 (71.93)	87 (76.32)
HR status, *n* (%)			
HR+	191 (63.67)	110 (64.33)	67 (58.77)
HR−	109 (36.33)	61 (35.67)	47 (41.23)
Metastases sites, *n* (%)			
aBC, HR+			
Lymph node	165 (55.00)	94 (54.97)	61 (53.51)
Adrenal gland	5 (1.67)	2 (1.17)	3 (2.63)
Bone	83 (27.67)	48 (28.07)	31 (27.19)
Digestive organs	62 (20.67)	35 (20.47)	24 (21.05)
Kidney	1 (0.33)	0 (0.00)	1 (0.88)
Nervous system	27 (9.00)	14 (8.19)	12 (10.53)
Ovary	2 (0.67)	2 (1.17)	0 (0.00)
Respiratory	47 (15.67)	26 (15.2)	19 (16.67)
Skin	17 (5.67)	9 (5.26)	7 (6.14)
Urinary organs	1 (0.33)	1 (0.58)	0 (0.00)
Other/unspecified	59 (19.67)	38 (22.22)	17 (14.91)
aBC, HR−			
Lymph node	72 (24.00)	38 (22.22)	33 (28.95)
Adrenal gland	5 (1.67)	2 (1.17)	3 (2.63)
Bone	41 (13.67)	18 (10.53)	22 (19.30)
Digestive organs	25 (8.33)	10 (5.85)	15 (13.16)
Kidney	0 (0.00)	0 (0.00)	0 (0.00)
Nervous system	26 (8.67)	10 (5.85)	15 (13.16)
Ovary	2 (0.67)	1 (0.58)	1 (0.88)
Respiratory	45 (15.00)	22 (12.87)	22 (19.30)
Skin	8 (2.67)	3 (1.75)	4 (3.51)
Urinary organs	2 (0.67)	1 (0.58)	1 (0.88)
Other/unspecified	47 (15.67)	22 (12.87)	24 (21.05)
Number of metastatic sites, mean (SD)			
All aBC	3.11 (1.62)	2.89 (1.6)	3.41 (1.6)
aBC, HR+	2.91 (1.57)	2.83 (1.62)	3.04 (1.54)
aBC, HR−	3.5 (1.63)	3.03 (1.56)	3.97 (1.55)
Number of metastatic sites, median (IQR)			
All aBC	3.0 (2.0, 4.0)	3.0 (2.0, 4.0)	3.0 (2.0, 4.0)
aBC, HR+	3.0 (2.0, 4.0)	3.0 (1.25, 4.0)	3.0 (2.0, 3.5)
aBC, HR−	3.0 (2.0, 4.0)	3.0 (2.0, 4.0)	4.0 (3.0, 5.0)

*Note:* HER2, human epidermal growth factor 2; IHC, immunohistochemistry.

Abbreviations: aBC, advanced breast cancer; HR, hormone receptor.

Outside of lymph nodes, the most common specified metastatic sites among HR+ patients were bone (HER2‐ultralow HR+ patients: 28%; no observable IHC staining HR+ patients: 27%) and digestive organs (HER2‐ultralow HR+ patients: 20%; no observable IHC staining HR+ patients: 21%). Bone was also the most common specified metastatic site among no observable IHC staining HR− patients (19%), whereas respiratory organs were the most common site among HER2‐ultralow HR− patients (13%).

### 3.5. Lines of Therapy

Most HER2‐ultralow patients (81.3%, n/N = 139/171) and patients with no observable IHC staining (86.0%, n/N = 98/114) received an anticancer drug on or after their aBC staging date. Table 4 presents treatment utilization for each cohort by regimen class (as defined in Supporting Table S5). Among HER2‐ultralow HR+ and HR− patients, 65.3% and 63.2% of LOT1 patients progressed to second LOT (LOT2), respectively. Among HR+ and HR− patients with no observable IHC staining, 52.3% and 60.0% of LOT1 patients progressed to LOT2, respectively. The most common LOT1 regimen among HR+ patients was hormone therapy (HT) alone (44%–48%), followed by CDK4/6 inhibitors with HT (CDK4/6 + HT) (22%–29%) and chemotherapy (14%–22%). Among HR− patients, the most common LOT1 regimens were chemotherapy (49%–61%) and immunotherapy‐based treatments (21%–34%).

**TABLE 4:** Lines of therapy by HER2/HR status and regimen class.

**LOT1 total**	**HER2-ultralow, HR+**	**No observable IHC staining, HR+**	**HER2-ultralow, HR−**	**No observable IHC staining, HR−**
	**101**	**63**	**38**	**35**

Anti‐HER2	1 (0.99%)	1 (1.59%)	2 (5.26%)	2 (5.71%)
CDK4/6 + HT	22 (21.78%)	18 (28.57%)	1 (2.63%)	1 (2.86%)
CDK4/6 w/o HT	1 (0.99%)	1 (1.59%)	0 (0%)	0 (0%)
Chemotherapy	22 (21.78%)	9 (14.29%)	23 (60.53%)	17 (48.57%)
HT alone	48 (47.52%)	28 (44.44%)	1 (2.63%)	3 (8.57%)
IO‐based	5 (4.95%)	5 (7.94%)	8 (21.05%)	12 (34.29%)
OT + HT	1 (0.99%)	0 (0%)	0 (0%)	0 (0%)
OT w/o HT	1 (0.99%)	0 (0%)	3 (7.89%)	0 (0%)
Sacituzumab	0 (0%)	1 (1.59%)	0 (0%)	0 (0%)

**LOT2 total**	**66**	**33**	**24**	**21**

Anti‐HER2	0 (0%)	2 (6.06%)	2 (8.33%)	0 (0%)
CDK4/6 + HT	7 (10.61%)	7 (21.21%)	0 (0%)	0 (0%)
CDK4/6 w/o HT	5 (7.58%)	1 (3.03%)	0 (0%)	0 (0%)
Chemotherapy	17 (25.76%)	10 (30.3%)	11 (45.83%)	14 (66.67%)
HT alone	24 (36.36%)	12 (36.36%)	1 (4.17%)	0 (0%)
IO‐based	5 (7.58%)	0 (0%)	6 (25%)	3 (14.29%)
OT + HT	5 (7.58%)	1 (3.03%)	0 (0%)	0 (0%)
OT w/o HT	2 (3.03%)	0 (0%)	2 (8.33%)	1 (4.76%)
Sacituzumab	1 (1.52%)	0 (0%)	2 (8.33%)	3 (14.29%)

**LOT3 total**	**36**	**24**	**19**	**15**

Anti‐HER2	2 (5.56%)	0 (0%)	1 (5.26%)	1 (6.67%)
CDK4/6 + HT	4 (11.11%)	4 (16.67%)	0 (0%)	0 (0%)
CDK4/6 w/o HT	1 (2.78%)	0 (0%)	0 (0%)	0 (0%)
Chemotherapy	8 (22.22%)	2 (8.33%)	11 (57.89%)	4 (26.67%)
HT alone	16 (44.44%)	14 (58.33%)	2 (10.53%)	0 (0%)
IO‐based	2 (5.56%)	1 (4.17%)	1 (5.26%)	6 (40%)
OT + HT	1 (2.78%)	3 (12.5%)	0 (0%)	0 (0%)
OT w/o HT	0 (0%)	0 (0%)	1 (5.26%)	2 (13.33%)
Sacituzumab	2 (5.56%)	0 (0%)	3 (15.79%)	2 (13.33%)

*Note:* HER2, human epidermal growth factor 2; IHC, immunohistochemistry; IO, immunotherapy; OT, other targeted treatments. The bolded values in the LOT1, LOT2, and LOT3 rows represent the total patient counts for each treatment line, categorized by their specific HER2 and HR status. These totals show a consistent decline in the number of patients across all four subgroups as they progress from the first line of therapy (LOT1) through the third (LOT3).

Abbreviations: HR, hormone receptor; HT, hormone therapy; LOT, line of therapy.

### 3.6. Outcomes

In the LOT1 setting, among HER2‐ultralow HR+ and HR− patients, the median TTF was 7.73 months (95% CI: 5.10–11.83) and 5.00 months (95% CI: 2.93–6.57) after initiating LOT1, respectively (Table 5). For HR+ and HR− patients with no observable IHC staining, the median TTF was 9.40 months (95% CI: 6.07–19.47) and 3.17 months (95% CI: 2.03–4.20) after initiating LOT1, respectively. While we observed shorter median TTFs after all LOTs for the HER2‐ultralow HR+ cohort compared to the no observable staining HR+ cohort, these differences did not reach statistical significance by log‐rank testing regardless of the LOT number (across LOT *p* − value > 0.05).

**TABLE 5:** Time to treatment failure (months) after each LOT, stratified by HER2/HR status.

	**HER2-ultralow, HR+**		**No observable IHC staining, HR+**		**HER2-ultralow, HR−**		**No observable IHC staining, HR−**	
	**N**	**Median (95% CI)**	**N**	**Median (95% CI)**	**N**	**Median (95% CI)**	**N**	**Median (95% CI)**

LOT1	87	7.73 (5.10, 11.83)	48	9.40 (6.07, 19.47)	33	5.00 (2.93, 6.57)	31	3.17 (2.03, 4.20)
LOT2	49	7.67 (4.63, 12.10)	27	10.73 (5.17, 13.07)	22	6.83 (3.27, 8.67)	18	4.33 (2.47, 10.30)
LOT3	31	7.63 (5.20, 13.97)	19	9.27 (3.63, 16.17)	15	5.83 (1.63, 9.00)	13	3.43 (1.10, 5.47)

*Note:* HER2, human epidermal growth factor 2; IHC, immunohistochemistry.

Abbreviations: HR, hormone receptor; LOT, line of therapy.

## 4. Discussion

The DESTINY‐Breast04 study established HER2‐low mBC as a clinically relevant category of HER2 expression with therapeutic implications [1]. The latest results from the DESTINY‐Breast06 trial indicate that patients with HR+ HER2‐ultralow mBC (who have previously received ≥ 2 lines of endocrine therapy with or without targeted therapy or experienced rapid disease progression after treatment with ≥ 1 line of adjuvant endocrine therapy) exhibit improvement in PFS when treated with T‐DXd compared to the current standard of care, and T‐DXd is now approved for the HER2‐ultralow BC patient population [2]. The 2025 NCCN Clinical Practice Guidelines in Oncology (NCCN Guidelines) reflect this new indication, listing fam‐T‐DXd‐nxki as a second‐line treatment option for HER2‐negative IHC1+ or 2+/ISH negative (NCCN Category 1, preferred) and IHC0+ (Category 2A, other recommended regimen) HR+ recurrent unresectable (local or regional) or Stage IV BC disease and may be used in those previously treated with ≥ 1 line of endocrine‐based therapy in the metastatic setting [12]. As such, incorporating the HER2‐low and HER2‐ultralow classifications into standard pathology practice is vital to ensuring patients receive treatment best suited to their needs. We conducted a retrospective cohort study using digitally scanned slides and EHR data from Mayo Clinic to investigate the prevalence of HER2‐ultralow status among the Mayo aBC/mBC patient population and to assess interobserver concordance of board‐certified pathologists re‐scoring 300 HER2‐stained aBC/mBC slides previously scored as IHC 0.

Despite the prognostic value of HER2 scoring in treatment decision‐making and patient outcomes, the limited number of studies assessing the reliability and reproducibility of HER2 IHC scoring among pathologists thus far show significant interpathologist discordance (particularly in cases with low staining). In a recent study by Zaakouk et al. (2023), pathologists scored 50 HER2 IHC slides from BC patients, and interobserver agreement was 0.41–0.8 within the HER2‐low subgroup [13]. Similarly, Baez‐Navarro et al. (2023) and Scott et al. (2021) also showed significant discordance in reported HER2 status between scored pathology samples [4, 14]. These findings underscore the need for more and effective training for accurate HER2 scoring, as discrepancies among pathologists can affect treatment options and outcomes for patients. Even more so, distinguishing the clinically relevant subtypes of HER2‐low cancers has not been well studied. However, as awareness increases regarding low levels of HER2 expression, concordance is beginning to improve; a concordance study on DESTINY‐Breast06 patients showed an overall concordance percentage of 77.8% for HER2‐low patients [15].

About three in five aBC patients with tumors originally classified as IHC 0 met the HER2‐ultralow criteria according to at least one pathologist in this study. A similar study performed in China found that among 1363 HER2‐negative patients, 481 (35.3%) were either HER2‐ultralow or had no observable IHC staining, and of these, the prevalence of HER2‐ultralow was 82% [16]. Furthermore, a DESTINY‐Breast06 concordance study found that of patients with tumors locally scored as IHC 0, 40% were re‐scored as HER2‐ultralow [15]. In these studies, a significant proportion of patients previously classified as IHC 0, even more than half in some cases, indeed met the criteria for HER2‐ultralow [14], indicating that a large population of patients may exist who could benefit from HER2‐targeted therapies such as T‐DXd who were not previously considered eligible for this treatment.

In addition, HER2‐ultralow HR+ patients exhibited shorter TTF in general and more so in later lines compared to patients with no observable IHC staining. While this difference was not statistically significant in this study, it may underscore a potential unmet need for more effective targeted treatment for this patient population, and therefore future studies with larger sample sizes and by BC stage are needed to further evaluate this finding.

Concordance between the two pathologists across any of the HER2 categories was moderate at best in the IHC 0 with no observable membrane staining and IHC 2+/indeterminate categories. Pathologists were also blinded to the goal of identifying HER2‐ultralow cases, though in theory, they could have deduced the study objective based on the fact that all the samples they were asked to review were IHC 0. Nevertheless, based on these results, it appears that no staining and greater levels of staining are more straightforward to classify than lower levels of staining, even among expert pathologists with BC‐specific expertise. With the addition of a third pathologist as a sensitivity analysis, agreement within each category generally rose but was still only fair among the HER2‐ultralow and IHC 1+ categories. These results are consistent with existing evaluations of concordance in HER2 scoring, which report that pathologists tend to disagree in differentiating IHC 0 versus IHC 1+ and IHC 1+ versus IHC 2+ [17, 18]. The relatively low concordance between pathologists in identifying lower levels of HER2 expression suggests the need for increased precision and standardization in determining IHC status. Tozbikian et al. (2024) emphasize the importance of adhering to best practices such as ensuring sample quality before IHC testing, using a standardized algorithm for scoring, and seeking consensus from other pathologists in more challenging cases [19]. They additionally recommend re‐scoring older biopsy samples previously scored as IHC 0 or 1+, so that patients with HER2‐ultralow staining receive the most up‐to‐date treatment recommendations. Promoting effective communication between pathologists and oncologists and providing appropriate training will be crucial in this effort. Finally, prior studies have shown that combining IHC scores results in increased concordance and score reproducibility, especially when differentiating no observable staining versus any staining [13, 14]. As such, refining and simplifying the HER2 scoring system may bolster concordance and help pathologists more easily identify patients who could benefit from targeted therapies.

A major strength of this study is the number of IHC 0 cases available for analysis with digitized pathology slides (*n* = 300). With this sample size, this study was able to provide a reasonable estimate of the prevalence of HER2‐ultralow among aBC/mBC patients, as well as a more in‐depth assessment of concordance and discordance between pathologists in differentiating the no observable IHC staining, HER2‐ultralow, and IHC 1+ categories specifically. Studies investigating the boundaries between these cut points are crucial as next‐generation HER2‐directed ADCs become more widely approved for the HER2‐low and HER2‐ultralow patient populations. Our use of the de‐identified Mayo Clinic EHR enables this robust analysis due to the availability of all patient records over the duration of their care at Mayo Clinic, including clinical notes, pathology reports, and laboratory testing. In addition, this study is the first of its kind in the US to estimate the prevalence of HER2‐ultralow. However, patients within the Mayo Clinic population are predominantly White, and findings from this cohort may not be generalizable to a nationally representative US population. Data from the Mayo Clinic EHR are also generated from a real‐world clinical setting, and no re‐staining of slides was done before digitization; the data, therefore, may be subject to miscoding, missing data, and errors (e.g., loss of antigenicity over time could have led to misclassification). Data for time periods where treatment was provided outside Mayo Clinic may be incomplete or missing. Finally, concordance was not assessed separately for borderline cases closer to 0% staining; intraobserver agreement was not evaluated, so this study does not consider the potential for one pathologist to score the same slide differently at different time points; and pathologists were not trained specifically to identify HER2‐ultralow patients, which may have affected the results. Specific education and training for HER2‐ultralow identification may boost concordance in this category.

Moving forward, as HER2‐low and HER2‐ultralow classifications become more widely accepted in the HER2 classification framework, technologies incorporating artificial intelligence (AI) such as computer vision may aid in automating and standardizing the IHC scoring process. This study showcased the use of digital pathology capabilities in facilitating pathologist review and highlighted the moderate concordance between scorers. A next step for this technology will be to develop models that can assign IHC scores to slide images. Such models have already been developed and demonstrate reasonable performance against a ground truth but cannot yet discriminate reliably between HER2 IHC 0 with no observable staining, HER2‐ultralow, and HER2 IHC 1+ samples [20, 21]. Combining AI with the expertise of human pathologist review may be an avenue to increase both the accuracy and efficiency of IHC scoring: a study of 246 IHC 0 and IHC 1+ cases found that AI‐assisted review of slides can increase the consistency and accuracy of scoring in these categories [22]. Expansion of HER2‐ultralow samples into such studies will aid in more effectively identifying patients who may benefit from targeted therapies such as T‐DXd.

## 5. Conclusion

About three in five aBC patients with tumors originally classified as HER2 IHC 0 met the HER2‐ultralow criteria by at least one pathologist, implying that a significant number of additional patients may benefit from HER2‐targeted therapy. The relatively low concordance between expert breast pathologists in identifying lower levels of HER2 expression suggests the need for increased enhanced training and review for practicing pathologists, precision enabled by AI‐assisted digital tools, and continuing to follow best practice recommendations in determining HER2 IHC status.

## Author Contributions

Conception and design: Sandhya Mehta, Michele Sue‐Ann Woo, Simone T. Sredni, Rosemarie Di Donato, Tyler Wagner, Jason Hipp, and Hannah Barman. Administrative support: Katherine Krieser, Elise Bieri Patzke, Natalie Kyek, Christian Anderson, Tyler Wagner, and Hannah Barman. Collection and assembly of data: Anushree R. Iyengar, Erinn P. Downs, Nivedita Rangarajan, Safak Simsek, Aziza Nassar, Darren M. Wilson, and Hannah Barman. Data analysis and interpretation: Anushree R. Iyengar, Erinn P. Downs, Sandhya Mehta, Nivedita Rangarajan, Safak Simsek, Aziza Nassar, and Hannah Barman.

## Funding

This study was sponsored by Daiichi Sankyo Inc. In March 2019, AstraZeneca entered into a global development and commercialization collaboration agreement with Daiichi Sankyo for trastuzumab deruxtecan (T‐DXd; DS‐8201).

## Disclosure

All authors wrote the manuscript, approved the final manuscript, and are accountable for all aspects of the work.

## Ethics Statement

Procurement of pathology slides was approved by the Mayo Clinic Institutional Review Board. All further analyses were conducted on completely de‐identified patient information and did not require IRB approval. This study was performed in accordance with the Declaration of Helsinki.

## Conflicts of Interest

Tyler Wagner and Nivedita Rangarajan are employees of nference, and nference received funding from Daiichi Sankyo, Inc. to conduct this research. Anushree R. Iyengar, Christian Anderson, Hannah Barman, and Safak Simsek were employees of nference at the time the study was conducted, and nference received funding from Daiichi Sankyo, Inc. to conduct this research. Sandhya Mehta and Michele Sue‐Ann Woo are employees of Daiichi Sankyo, Inc. Simone T. Sredni and Rosemarie Di Donato are employees of AstraZeneca. Michele Sue‐Ann Woo owns stock in Daiichi Sankyo, Inc. Jason Hipp is a consultant for Noetik and owns stock. The remaining authors declare no conflicts of interest.

## Supporting Information

Additional supporting information can be found online in the Supporting Information section.

## Supporting information


**Supporting Information** Table S1. Structured diagnosis codes for breast cancer and secondary neoplasms. Table S2. NLP synonyms for breast cancer diagnosis.Table S3. Augmented curation model performance metrics. Table S4. Curated drug list for treatment pattern characterization. Table S5. Drug regimen classifications for treatment pattern characterization.

## Data Availability

The data that support the findings of this study are derived from the Mayo Clinic EHR and are available under license from the Mayo Clinic and nference. The data used for this study cannot be shared publicly as the sharing of individuals’ protected health information (PHI) is forbidden.
