# Supplementary material for: HER2‐Ultralow: Prevalence, Characteristics, and Treatment Choices Among Advanced Breast Cancer Patients With Tumors Initially Scored as IHC 0
Source: Breast J. 2026 Jun 24;2026:7212120. doi: 10.1155/tbj/7212120 (PMC13291886; doi:10.1155/tbj/7212120)
Supplement: Supplementary file 1 — Supporting Information Table S1. Structured diagnosis codes for breast cancer and secondary neoplasms. Table S2. NLP synonyms for breast cancer diagnosis.Table S3. Augmented curation model performance metrics. Table S4. Curated drug list for treatment pattern characterization. Table S5. Drug regimen classifications for treatment pattern characterization. [file TBJ-2026-7212120-s001.docx]

**Table S1. Structured diagnosis codes for breast cancer and secondary neoplasms**

| **Indication** | **ICD-9** | **ICD-10** | **SNOMED** |
| --- | --- | --- | --- |
| Breast cancer | 174.0, 174.1, 174.2, 174.3, 174.4, 174.5, 174.6, 174.8, 175.0, 175.9, 198.81, 233.0 | С44.501, С44.521, С44.591, С50, С50.011, С50.012, С50.019, С50.021, С50.022, С50.029, С50.11, С50.111, С50.112, С50.119, С50.121, С50.122, С50.129, С50.211, С50.212, С50.219, С50.221, С50.222, С50.311, С50.312, С50.319, С50.321, С50.322, С50.411, С50.412, С50.419, С50.421, С50.422, С50.429, С50.511, С50.512, С50.519, С50.521, С50.522, С50.611, С50.612, С50.619,, С50.621, С50.622, С50.811, С50.812, С50.819, С50.821, С50.822, С50.9, С50.91, С50.911, С50.912, C50.919, C50.921, C50.922, C50.929, C79.81, C84.70, D05.00, D05.01, D05.02, D05.10, D05.11, D05.12, D05.80, D05.81, D05.82, D05.90, D05.91, D05.92 | 1740000, 1740110, 1740112, 1740113, 1740120, 1740121, 1740131, 1741111, 1983110, 1983130, 1983160, 137204015, 143635016, 156065013, 156068010, 156355012, 174574016, 174575015, 174577011, 174578018, 200714014, 201290013, 216211016, 289137018, 289141019, 289142014, 289143016, 289144010, 289145011, 289146012, 289153015, 290868010, 379661016, 379662011, 379663018, 379665013, 379666014, 411827018, 414829016, 426379013, 459377012, 459457018, 510640013, 510995017, 1210642019, 1210672013, 1210673015, 1211597012, 2157601016, 2160190012, 2164001014, 2164002019, 2618556017, 2618557014, 2643561013, 2643562018, 2643563011, 2643564017, 2841818010, 2870872019, 2871750016, 2871914019, 2871985014, 2902359012, 2916990012 |
| Secondary neoplasms of lymph nodes | - | C77.0, C77.1, C77.2, C77.3, C77.4, C77.5, C77.8, C77.9 | - |
| Secondary neoplasms of other sites | - | C78.00, C78.01, C78.02, C78.1, C78.2, C78.30, C78.39 C78.4, C78.5, C78.6, C78.7, C78.80, C78.89, C79.00, C79.01, C79.02, C79.10, C79.11, C79.19, C79.2, C79.31, C79.32, C79.40, C79.49, C79.51, C79.52, C79.60, C79.61, C79.62, C79.63, C79.70, C79.71, C79.72, C79.82, C79.89, C79.9 | - |

**Table S2. NLP synonyms for breast cancer diagnosis**

| **Condition** | **Synonyms** |
| --- | --- |
| Breast cancer | metastatic_breast_carcinoma, ductal_carcinoma_in_situ, node_positive_breast_cancer, lobular_carcinoma_stage_iii, advanced_breast_cancer_patients, intraductal_papillary_adenocarcinoma_with_invasion, basal_breast_cancer, breast_malignant_lymphoma, non_malignant_breast_tissue, lobular carcinoma, her2_negative_breast_cancer, breast invasive_lobular_carcinoma, stage_ii_breast_cancer, luminal_b_breast_cancer, breast_cancer metastatic_disease, tubular_breast_cancer_stage_i, infitrating_ductal_carcinoma_of_upper_outer_quadrant_of_left_female_breast, invasive_papillary_breast_carcinoma, metastatic squamous_cell breast_carcinoma, mixed_acinar_ductal_carcinoma, microinvasive_ductal_carcinoma, breast_adenocarcinoma, unilateral_breast_cancer, stage_ia_breast_cancer, stage_iv_breast_cancer, breast_cancer_lymphedema, invasive_ductal_and_lobular_carcinomas, classic_lobular_carcinoma_in_situ, breast_adenocarcinoma_stage_i, malignant_neoplasm_of_lower_inner_quadrant_of_female_breast, mucinous_breast_carcinoma, breast_ovarian_cancer_syndrome, lobular_carcinoma _in_situ_with_ microinvasion, squamous_cell_carcinoma_of_the_breast, stage _b_breast _carcinoma, infiltrating_carcinoma_with_ductal_and_lobular_features, invasive_ductal _carcinoma_not _otherwise_specified, infiltrating_ductal_carcinoma_of_lower_outer_quadrant_of_left_female_breast, stage_iv _inflammatory_breast_carcinoma, basal_breast_carcinoma, pagets_disease _and _intraductal_carcinoma_of _breast, malignant_melanoma_of_skin_of_breast, breast_adenocarcinoma_stage_ill, intraductal _micropapillary_carcinoma, primary_malignant_neoplasm_of_central portion_of female _breast, cancer_of _upper_breast, invasive _ductal _breast _carcinoma_stage_ii, infiltrating_lobular_carcinoma_of_left_female_breast, adenocarcinoma_of_mammary_gland_type, infiltrating_lobular_carcinoma_of_right_female_breast, comedocarcinoma_of_breast, carcinoma_in_situ_of_the_breast, progesterone_receptor_negative_breast_cancer, contralateral_breast _cancer, lobular_breast_carcinoma, intraductal_carcinoma_in_situ_of_left_breast, metastatic_lobular_breast_carcinoma, pleomorphic_lobular _carcinoma, stage _iii_breast_carcinoma, estrogen_receptor_negative_breast_cancer, female_breast_carcinoma, stage _jib_ inflammatory_breast_carcinoma, recurrent_inflammatory_breast_carcinoma, invasive_breast_ductal_carcinoma, hereditary_breast _cancer_syndromes, breast_myoepithelial _carcinoma, infiltrating_duct_carcinoma_of_left_female_breast, breast_carcinoma_metastatic_in_the_liver, anaplastic _breast_carcinoma, clinging_ductal _carcinoma_in_situ, invasive_mixed_breast_carcinoma, invasive_apocrine_breast_carcinoma, lobular_carcinoma_recurrent, breast_invasive_ductal_carcinoma, ductal carcinoma, stage_i_breast_cancer, breast_adenocarcinoma_metastatic, noninvasive_ductal_carcinoma, contralateral_breast_carcinoma, breast _adenoid _cystic_carcinoma, metastatic_ductal_breast_carcinoma, mammary_gland_cancer, hereditary_breast_ovarian_cancer_brca1, metaplastic_breast_carcinomas, locally_advanced _breast_cancer, lobular_breast_cancer, primary_cancer_of_breast, breast_invasive_carcinoma, er_positive_breast_cancer, advanced _triple_negative_breast_cancer, stage_iia_breast_carcinoma, advanced_breast_cancer_2, invasive_pleomorphic_lobular_carcinoma, medullary_carcinoma_of_the_breast, sporadic_breast_cancer, cancer_of_breast_stage_iib, synchronous_bilateral_breast_carcinoma, occult breast carcinoma, stage _ilic_breast_cancer_ajcc_v7, intraductal_carcinoma_and_lobular_carcinoma_in_situ, minimally_invasive_breast_cancer, primary_malignant_neoplasm_of_female_breast, intraductal_papilloma_with _ductal_carcinoma_in_situ, malignant_neoplasm_of_axillary_tail_of_female_breast, pleomorphic_variant_of_invasive_lobular_carcinoma, breast_adenocarcinoma_stage_iv, hormone_refractory_breast_cancer, grade_1_ invasive_breast_carcinoma, mucinous_breast_cancer_stage_i, breast_adenocarcinoma_recurrent, papillary_breast_carcinoma _in _situ, infiltrating_ductal_carcinoma_of_upper_inner_quadrant_of_eft_female_breast, brca2 _hereditary_breast_and _ovarian_cancer_syndrome, multifocal_breast _cancer, bilateral_breast _cancer, invasive_breast _carcinoma, breast _phyllodes_tumors, pleomorphic_invasive_lobular_carcinoma, mammary _analogue_secretory_carcinoma, lobular_carcinoma_ in_situ_of_right _breast, overlapping_cancer_of_female_breast, prostate _ductal_adenocarcinoma, hereditary_breast_ovarian_cancer, gestational_breast_cancer, pikca_mutated_breast_cancer, infiltrating_duct_carcinoma_of_breast, infiltrating_duct_carcinoma_of_right_female_breast, locoregionally_recurrent_breast_cancer, invasive_papillary_breast_cancer, breast_carcinoma_metastatic_in_the_skin, apocrine_breast_carcinoma, infiltrating_duct_carcinoma_of_female_breast, malignant_mammary_tumors, malignant_neoplasm_of_breast_stage_i, ductal_breast_carcinoma_stage_ili, triple_negative_breast_cancer, malignant_neoplasm_of_breast_upper_outer_quadrant, invasive_ductal_cancer, carcinoma_in_situ _of_right_breast, hereditary_breast_and _ovarian_cancer_syndrome, mammary_ductal_carcinoma, ductal _carcinoma_in_situ_with_microinvasion, male_breast_carcinoma, breast_adenocarcinoma_stage_ii, advanced_her2_negative_breast_cancer, lobular_breast_carcinoma_stage_i, her2_receptor_negative_breast_cancer, recurrent_invasive_ductal_breast_carcinoma, lobular_breast_carcinoma_stage_ili, papillary_carcinoma_of_the_breast, her2_positive_breast_cancer, invasive_ductal_breast_carcinoma, female_breast_cancer, inflammatory_breast_cancer, male_breast_cancer, grade_3_invasive_breast_carcinoma, infiltrating_duct_and_lobular_carcinoma, breast_cancer_visceral_crisis, lobular_carcinoma_stage_iv, alcohol_and_breast_cancer, invasive_ductal _breast_carcinoma_stage_iii, invasive_ductal_breast_carcinoma_stage_i, early_stage_breast_carcinoma, breast_cancer, lobular_carcinoma_in_situ, progesterone receptor _positive breast cancer, hormone_receptor_positive_breast_cancer, malignant_neoplasm_of_upper_outer_quadrant_of_female_breast, premenopausal_breast_cancer, metastatic invasive_lobular_carcinoma, stage _ib_breast_cancer_ajcc_v7, mucinous_breast_cancer, intraductal_papillary_carcinoma, lobular_carcinoma_in_situ_of_left_breast, hormone _receptor_positive_breast_carcinoma, familial_breast_ovarian_cancer, medullary_breast_carcinoma, grade_2_invasive_breast_carcinoma, primary_invasive_malignant_neoplasm_of_female_breast, atypical_medullary_breast_cancer, invasive_intraductal_papillary_mucinous_carcinoma, mucoepidermoid_ carcinoma_of_the_breast, infiltrating_ductal_carcinoma_of_upper_outer_quadrant_of_right_female_breast, carcinoma _of_breast_upper_outer_quadrant, mixed _ductal_endocrine_carcinoma, subsequent_invasive_breast_cancer, her2_positive_advanced_breast _cancer, ductal_breast_carcinoma_stage_iv, node_negative_breast_cancer, metastatic_ invasive _ductal _carcinoma, intraductal_carcinoma_in_situ_of _right_breast, breast_cancer_3, invasive_lobular_breast_cancer, malignant_neoplasm_of_breast_upper_inner_quadrant, advanced_stage_breast_cancer, familial _breast_cancer, tubular_breast_cancer, triple_positive_breast _cancer, mammary_adenocarcinoma, lobular_carcinoma_stage_i, recurrent_metastatic_breast_carcinoma, stage_iib_breast_carcinoma, mixed _lobular _and_ductal_breast_carcinoma, ductal _carcinoma in_situ_solid _type, ductal invasive_carcinoma, brea_mutated_breast_cancer, noninfiltrating_intraductal_carcinoma, invasive_ductal_carcinoma_with_an_extensive_intraductal_component, malignant_neoplasm_of_upper_inner_quadrant_of_female_breast, infiltrating_lobular_mixed_with_other_types_of_carcinoma, mammary_gland_carcinoma, basal _like_breast_carcinomas, intraductal_breast_cancer_in_situ, apocrine _intraductal_carcinoma, metastasis_from_malignant_tumor_of_breast, microinvasive _breast_carcinoma, invasive_micropapillary_breast_cancer, colloidal _breast_carcinoma, breast_carcinoma_metastatic_ in_the_brain, borderline_breast_phyllodes_tumor, solid_papillary_carcinoma_of_the_breast, solid_papillary_carcinoma_in_situ_of_breast, childhood_breast_carcinoma, scirrhous_breast_carcinoma, local_recurrence_of_malignant_tumor_of_breast, pleomorphic_lobular_breast_carcinoma_in_situ, malignant_myoepithelioma_of_the_breast, metastatic_breast_cancer, invasive_lobular_cancer, estrogen_receptor_positive_breast_cancer, advanced_breast_cancer, metastatic_triple_negative_breast_carcinoma, metastatic_recurrent_breast_cancer, stage_ilia_breast_carcinoma, luminal_breast_cancer, breast_carcinoma_metastatic_in_the_bone, carcinoma_in_situ_of_left_breast, pleomorphic_lobular_carcinoma_in_situ, lobular_breast_carcinoma_stage_ii, lobular_carcinoma_stage_ii, her2_positive_carcinoma_of_breast ductal_carcinoma_in_situ_category, malignant_neoplasm_of_lower_outer_quadrant_of_female_breast, intracystic_papillary_breast_carcinoma, tubulolobular_carcinoma, breast_carcinoma_metastatic_in_the_lung, carcinoma_in_situ_of_female_breast, atypical_medullary_breast_carcinoma, refractory_breast_carcinoma, breast_large_cell_neuroendocrine_carcinoma, cancer_of_midline_of_breast, malignant_neoplasm_of_axillary_tail_of_breast, infiltrating_ductal_carcinoma_of_central_portion_of_left_female_breast, ductal_breast_carcinoma_stage_i, recurrent breast_cancer, breast_carcinoma, recurrent_metastatic_breast_cancer, breast_ductal_carcinoma, stage_iii_breast_cancer, invasive_ductal_cell_carcinoma, high _grade_invasive_ductal_carcinoma, breast_invasive_ductal_cancer, malignant_neoplasm_of_nipple_and_areola_of_male_breast, ductal_carcinoma_invasive, multicentric_breast_cancer, postmenopausal_breast_cancer, early_stage _invasive_breast_cancer, primary_invasive_breast_cancer, breast_small_cell_carcinoma, malignant_neoplasm_of_nipple_and_areola_of_female_breast, malignant_neoplasm_of_breast_stage_iv, recurrent lobular breast carcinoma, pleomorphic_breast_carcinoma, secretory_breast_carcinoma, invasive_micropapillary_breast_carcinoma, her_2_positive_advanced_breast_cancer |

**Table S3. Augmented curation model performance metrics**

| **Model Name** | **Precision** | **Recall** | **F1-score** |
| --- | --- | --- | --- |
| Disease Diagnosis | 0.92 | 0.92 | 0.92 |
| Cancer Staging | 0.97 | 0.97 | 0.97 |
| Oncology Biomarker | 0.94 | 0.93 | 0.94 |
| IHC Score Regex | 1.00 | 1.00 | 1.00 |

**Table S4. Curated drug list for treatment pattern characterization**

| **Drug class** | **Drug name** | **Drug synonyms** |
| --- | --- | --- |
| **Anti-HER2** | enhertu | enhertu, trastuzumab deruxtecan, fam-trastuzumab deruxtecan-nxki, ds-8201, ds-8201a, tdxd, t_dxd, t-dxd |
|  | herceptin | herceptin, herceptin hylecta, herzuma, kanjinti, ontruzant, perjeta-herceptin, phesgo, trazimera, hyaluronidase-oysk, rhumab her2, trastuzumab-anns, trastuzumab-dkst, trastuzumab-dttb, trastuzumab-pkrb, trastuzumab-qyyp, 4d5v8, abp 980, abp-980, dmb-3111, eg-12014, eg12014, r-597, sb-3, syd-977, syd977 |
|  | kadcyla | kadcyla, t dm1, trastuzumab emtansine, ado trastuzumab emtansine, trastuzumab dm1, trastuzumab mcc dm1, ado trastuzumab, t_dm1, t-dm1, tdm1, trastuzumab-mcc-dm1, pro-132365, pro132365, rg-3502 |
|  | lapatinib | tykerb, tyverb, lapatinib, lapatinib ditosylate, gsk-572016, gw 572016, gw-572016x, gw572016 |
|  | margetuximab | margenza, margetuximab, margetuximab-cmkb, mgah-22, mgah22 |
|  | perjeta | perjeta, pertuzumab, omnitarg, perjeta-herceptin, phesgo, 2c4 antibody, moab 2c4, monoclonal antibody 2c4, rhumab-2c4, r1273, rg-1273 |
|  | tukysa | tukysa, tucatinib, arry 380, arry-380, ont 380, ont-380 |
| **CDK4/6 inhibitor** | abemaciclib | abemaciclib, verzenio, ly-2835219, ly2835219 |
|  | palbociclib | palbociclib, ibrance, pd 0332991, pd 332991, pd-0332991, pd-332991, pd0332991, pd332991 |
|  | ribociclib | ribociclib, kisqali, kisqali femara co-pack, lee-011, lee-011a, lee011, lee011a |
| **Chemotherapy** | 5-fluorouracil (5fu) | 5-fluorouracil, actikerall, carac, efudex, fluoroplex, tolak, fluorouracil, nsc-19893, ro 2-9757 |
|  | acalabrutinib | acalabrutinib, calquence |
|  | azacitidine | azacitidine |
|  | belinostat | belinostat, beleodaq |
|  | bendamustine | bendamustine, bendeka, treanda |
|  | bortezomib | bortezomib, velcade |
|  | busulfan | busulfan, busulfex, myleran |
|  | cabozantinib | cabozantinib, cabometyx |
|  | capecitabine | capecitabine, xeloda, ecansya, r340, ro 09-1978/000, ro-09-1978/000 |
|  | carboplatin | carboplatin, paraplatin, nsc-241240 |
|  | carfilzomib | carfilzomib, kyprolis |
|  | carmustine | carmustine, gliadel wafer, bicnu |
|  | cisplatin | cisplatin |
|  | cyclophosphamide | cyclophosphamide, procytox, b 518, nsc 26271 |
|  | cytarabine | cytarabine |
|  | dabrafenib | dabrafenib, tafinlar |
|  | dasatinib | dasatinib, sprycel |
|  | decitabine | decitabine |
|  | docetaxel | docetaxel, taxotere, ckd-810, rp-6976 |
|  | doxorubicin | doxorubicin, adriamycin, doxil, myocet, hydroxydaunorubicin |
|  | epirubicin | epirubicin, ellence, pharmorubicin pfs, epiadriamycin, nsc-256942 |
|  | eribulin | eribulin, halaven, er-086526, eribulin mesylate |
|  | etoposide | etoposide, etopophos, toposar |
|  | fludarabine | fludarabine |
|  | gemcitabine | gemcitabine, gemzar, infugem, ly-188011, ly188011 |
|  | hydroxyurea | hydroxyurea |
|  | idarubicin | idarubicin, idamycin pfs |
|  | ifosfamide | ifosfamide, ifex |
|  | imatinib | imatinib, gleevec |
|  | irinotecan | irinotecan, onivyde, camptosar |
|  | ixabepilone | ixabepilone, ixempra, aza-epothilone b, azaepothilone b, bms 247550-01, bms-247550 |
|  | lurbinectedin | lurbinectedin, zepzelca |
|  | melphalan | melphalan, evomela |
|  | methotrexate | methotrexate, metoject, nordimet, otrexup, rasuvo, reditrex, trexall, xatmep, amethopterin, cl 14377, cl-14377, emt 25299, emt-25299, nsc-740, r 9985, r-9985 |
|  | methoxsalen | methoxsalen, oxsoralen-ultra |
|  | mitomycin | mitomycin |
|  | nilotinib | nilotinib, tasigna |
|  | olaratumab | olaratumab, lartruvo |
|  | oxaliplatin | oxaliplatin |
|  | paclitaxel | paclitaxel, abraxane, taxol, abi-007, bms 181339-01, bms-181339-01, dhp 107, mbt 0206, mbt-0206, nk 105, nsc-125973, qw-8184, s-8184 |
|  | pemetrexed | pemetrexed, alimta |
|  | romidepsin | romidepsin, istodax |
|  | temsirolimus | temsirolimus, torisel |
|  | thiotepa | thiotepa, tepadina |
|  | topotecan | topotecan, hycamtin |
|  | trametinib | trametinib, mekinist |
|  | vinblastine | vinblastine, vinblastine sulfate, nsc-47842 |
|  | vincristine | vincristine, marqibo, vincasar pfs |
|  | vinorelbine | vinorelbine, navelbine |
|  | vismodegib | vismodegib, erivedge |
| **Hormone therapy** | anastrozole | anastrozole, arimidex, anastrozol, ici d1033, ici-d1033, zd-1033, zd1033 |
|  | degarelix | degarelix, firmagon kit w diluent syringe, firmagon |
|  | enzalutamide | enzalutamide, xtandi |
|  | exemestane | exemestane, aromasin, fce-24304, fce24304, pnu-155971 |
|  | fulvestrant | fulvestrant, faslodex, ici 182,780, ici 182780, ici-182780, zd-9238, zd9238 |
|  | letrozole | letrozole, femara, kisqali femara co-pack, letrozol, cgs 20267, cgs-20267 |
|  | tamoxifen | soltamox, tamoxifen, tamoxifen citrate, nolvadex-d, ici 47699, ici-47699 |
|  | toremifene | toremifene, fareston, gtx-006, j33.157k |
|  | triptorelin pamoate | triptorelin pamoate, triptodur |
| **Immunotherapy** | aldesleukin | aldesleukin |
|  | atezolizumab | atezolizumab, tecentriq, mpdl 3280a, mpdl-3280a, mpdl3280a, rg-7446, rg7446 |
|  | bevacizumab | bevacizumab, avastin |
|  | cemiplimab | cemiplimab, libtayo |
|  | cetuximab | cetuximab, erbitux |
|  | daratumumab | daratumumab, darzalex |
|  | durvalumab | durvalumab, imfinzi |
|  | elotuzumab | elotuzumab, empliciti |
|  | gemtuzumab | gemtuzumab, mylotarg |
|  | interferon alfa-2b | intron a, interferon alfa-2b |
|  | ipilimumab | ipilimumab, ipi |
|  | nivolumab | nivolumab, opdivo |
|  | obinutuzumab | obinutuzumab, gazyva |
|  | pembrolizumab | pembrolizumab, keytruda, lambrolizumab, merck 3475, mk 3475, mk-3475, mk3475, sch 900475, sch-900475 |
|  | rituximab | rituximab, rituxan |
| **Other targeted** | alemtuzumab | alemtuzumab, campath, lemtrada |
|  | alpelisib | alpelisib, piqray, vijoice, byl 719, byl-719, byl719, nvp-byl719 |
|  | copanlisib | copanlisib, aliqopa, bay-80-6946, bay80-6946 |
|  | everolimus | everolimus, afinitor, votubia, zortress |
|  | gilteritinib | gilteritinib, xospata |
|  | ibrutinib | ibrutinib, imbruvica |
|  | idelalisib | idelalisib, zydelig, cal 101, cal-101, cal101, gs 1101, gs-1101, gs1101 |
|  | larotrectinib | larotrectinib, vitrakvi |
|  | lenvatinib | lenvatinib, lenvima |
|  | midostaurin | midostaurin, rydapt, tauritmo |
|  | neratinib | nerlynx, neratinib, hki-272, neratinib maleate, neratinib hki 22, hki22 |
|  | niraparib | zejula, niraparib |
|  | olaparib | olaparib, lynparza, azd 2281, azd-2281, azd2281, ku-0059436, ku-59436, ku59436 |
|  | osimertinib | osimertinib, tagrisso |
|  | panitumumab | panitumumab, vectibix |
|  | ramucirumab | ramucirumab, cyramza |
|  | ruxolitinib | ruxolitinib, jakafi |
|  | sacituzumab govitecan | sacituzumab govitecan-hziy, sacituzumab govitecan, trodelvy, hrs 7sn38, hrs7-sn38, immu 132, immu-132 |
|  | talazoparib | talazoparib, talzenna, talazoparib tosylate, bmn 673, bmn-673, lt-673 |
|  | umbralisib | umbralisib, ukoniq, rp-5264, tgr 1202, tgr-1202 |
| **Chemotherapy + immunotherapy** | polatuzumab vedotin | polatuzumab vedotin |

**Table S5. Drug regimen classifications for treatment pattern characterization**

| **Regimen class** | **Definition** |
| --- | --- |
| **Hormone therapy (HT) alone** | Hormone therapy with no other drugs |
| **CDK4/6 inhibitor with HT** | Contains CDK4/6 inhibitor with HT May include chemotherapy, immunotherapy, other targeted drugs |
| **CDK4/6 inhibitor without HT** | Contains CDK4/6 inhibitor without HT May include chemotherapy, immunotherapy, other targeted drugs |
| **Chemotherapy** | Chemotherapy alone Chemotherapy with HT Chemotherapy with HT and immunotherapy |
| **Immunotherapy-based regimen** | Immunotherapy alone Immunotherapy with chemotherapy (no HT) Immunotherapy with HT |
| **Other targeted with HT** | Other targeted with HT Other targeted with HT and chemotherapy Other targeted with HT and immunotherapy |
| **Other targeted without HT** | Other targeted alone Other targeted with immunotherapy and/or chemotherapy (no HT) |
| **Anti-HER2** | Contains any Anti-HER2 drug, regardless of other drugs in regimen |
| **Sacituzumab** | Contains sacituzumab govitecan, regardless of other drugs in regimen |
